# Androgen Receptor Signaling Inhibition in Advanced Castration Resistance Prostate Cancer: What Is Expected for the Near Future?

**DOI:** 10.3390/cancers14246071

**Published:** 2022-12-09

**Authors:** Javier Pozas, Sara Álvarez Rodríguez, Víctor Albarrán Fernández, Javier Burgos, Matteo Santoni, Ray Manneh Kopp, Javier Molina-Cerrillo, Teresa Alonso-Gordoa

**Affiliations:** 1 Medical Oncology Department, Hospital Universitario Ramón y Cajal, 28034 Madrid, Spain; 2 Urology Department, Hospital Universitario Ramón y Cajal, 28034 Madrid, Spain; 3 The Ramon y Cajal Health Research Institute (IRYCIS), CIBERONC, 28034 Madrid, Spain; 4 Medicine School, Alcalá University, 28805 Madrid, Spain; 5 Medical Oncology Department, Mazerata Hospital, 62100 Macerata, Italy; 6 Sociedad de Oncología y Hematología del Cesar, Valledupar 200001, Colombia

**Keywords:** androgen receptor, prostate cancer, resistance mechanisms, androgen receptor signaling inhibitors, antiandrogens

## Abstract

**Simple Summary:**

Prostate cancer progression is mainly driven by the androgen receptor (AR) signaling pathway and its inhibition has been the cornerstone in the treatment of patients harboring this disease. However, during the course of prostate cancer, various resistance mechanisms have been defined as responsible for tumor progression and the lack of response to current antiandrogen drugs. These resistance mechanisms act directly over the AR signaling pathway, but also over other bypassing pathways that could potentially be targetable. Therefore, the improvement in the molecular knowledge of prostate cancer progression and the development of new active therapies may overcome the current limits in prolonging the survival of patients with prostate cancer.

**Abstract:**

The androgen signaling pathway is the cornerstone in the treatment of high risk or advanced prostate cancer patients. However, in recent years, different mechanisms of resistance have been defined in this field, limiting the efficacy of the currently approved antiandrogen drugs. Different therapeutic approaches are under research to assess the role of combination therapies against escape signaling pathways or the development of novel antiandrogen drugs to try to solve the primary or acquired resistance against androgen dependent or independent pathways. The present review aims to summarize the current state of androgen inhibition in the therapeutic algorithm of patients with advanced prostate cancer and the mechanisms of resistance to those available drugs. In addition, this review conducted a comprehensive overview of the main present and future research approaches in the field of androgen receptor inhibition to overcome these resistances and the potential new drugs under research coming into this setting.

## 1. Introduction

Prostate cancer is the second most common solid tumor diagnosed in men with an estimated incidence for 2020 of 1.4 million new cases worldwide. Therefore, patients diagnosed with prostate cancer represent a major health clinical concern, and research focused on developing early and precise diagnostic tools as well as optimal treatment approaches is key [1]. Different clinical settings have been defined in prostate cancer that harbor a particular management according to their distinct prognosis and approved therapies. In the advanced disease, androgen deprivation therapy has been a cornerstone in the management of these patients. Unfortunately, patients will finally progress at some point of this therapy and new drugs have been developed to improve the oncological results by inhibiting the androgen receptor pathway more effectively, or by targeting bypassing pathways [1,2,3]. During the last years, the improvement in the molecular characteristics of prostate cancer has increased the relevance for a biomarker-driven therapeutic approach and, in this sense, the role of continuing AR signaling during the oncological progression as a key pathway [1]. Thus, deepening the knowledge in AR upstream and downstream signaling as well as potential targets with antitumoral activity is a constant field of research to keeping the blockade of the ARSI beyond the already AR signaling inhibitor (ARSI) approved drugs such as enzalutamide, apalutamide, darolutamide, or abiraterone acetate.

Overall, the aim of this review is to provide molecular insights on AR signaling in metastatic castration resistance prostate cancer (mCRPC) as well as current and future perspectives on drug development to adequately inhibit this key pathway and overcome potential resistance mechanisms to, finally, improve the patient survival. For this purpose, the relevant literature was searched through an automatized search in the PubMed bibliographic database published between 2000 and 2022 using the combination terms: “prostate cancer” OR “androgen receptor” OR “castration resistance” OR “metastatic prostate cancer” OR “antiandorgens”, “novel hormone agents”, “androgen receptor signaling inhibitors” OR “resistance mechanisms”.

## 2. Molecular Characterization of Metastatic Castration Resistance Prostate Cancer Focusing on Androgen Receptor Signaling Pathway

In an effort to understand the molecular landscape of mCRPC, Robinson and colleagues conducted a whole exome and transcriptome sequencing from 150 tumor samples of patients with mCRPC [1]. A driver genomic aberration was identified in almost all cases, the most prevalent being alterations in *AR*, suggesting that most mCRPC are dependent on AR signaling. The AR gene is located on chromosome X and consists of eight exons that encode for the AR nuclear receptor, a member of the steroid hormone nuclear receptor family [1,2,3]. AR has four functional regions: an NH2 terminal transactivation domain (NTD), a DNA-binding domain (DBD), a hinge region, and a C-terminal ligand binding domain (LBD). Given its ubiquitous distribution, it exerts a diverse range of biological actions including the development and regulation of the reproductive, cardiovascular, immune, neural, and hemopoietic systems.

In particular, AR signaling plays a major role in the development and homeostasis of the prostate [2]. In normal prostatic tissue, epithelial AR provides the prostate gland with several secretory proteins including prostate-specific antigen (PSA) and stromal AR facilitates prostate growth. Stromal AR-knockout mice have reduced epithelium proliferation, extracellular matrix remodeling, immune cell infiltration, and angiogenesis [3]. AR accumulates in the cytoplasm and is bound to a heat shock protein, cytoskeletal proteins, and other chaperones. Their role is to preserve AR in a conformation that allows for ligand binding and also to protect it from proteolysis.

Testosterone is produced primarily by the testes with a small contribution from the adrenal glands. 5α-Reductase is an enzyme that converts testosterone to DHT and is widely expressed within the prostate [4]. AR is activated after the binding of testosterone or 5 alpha dihydrotestosterone, which induces a conformational change in AR, facilitating interaction between the NTD and the LBD, which results in the dimerization of AR. The receptor is subsequently translocated into the nucleus [5]. There, the DBD tethers the AR to the promoter and enhancer regions of targeted genes through zinc fingers [6]. Once bound to the DNA, the AR dimer forms a complex with coregulatory proteins including SRC1, SRC2, SRC3, and p300/CBP, among others. Of note, the p160 coactivator complex formed by SRC1, TIF2, and SRC3 interacts with the NTS and the LBD, enhancing ligand-independent AR-mediated transcription of the target genes [7]. AR regulates the expression of genes that play an important role in prostate cancer such as KLK3, KLK2, TMPRSS2-ERG, IGFR-1, FKBP5, FOXp1, TACC2, and UGT1A1 [8]. It has been well-established that AR is the backbone of prostate cancer tumorigenesis by facilitating cell survival, proliferation, migration, and invasion [9]. Several decades ago, Huggins and Hodges demonstrated the sensitivity of prostate cancer to androgen deprivation [10]. Indeed, ADT has been the cornerstone therapy in metastatic prostate cancer ever since. Nonetheless, over time, most tumors develop mechanisms that enable them to escape ADT. It appears that genomic aberrancies affecting AR signaling are responsible for resistance to treatment. Interestingly, despite low levels of serum testosterone, AR signaling persists in the castration-resistance setting. In other words, ADT-refractory prostate cancer remains androgen-driven [11], probably due to activating AR mutations or amplifications that result in increased protein expression. The clinical proof-of-principle would be that novel antiandrogens abiraterone and enzalutamide are active in mCRPC. However, both intrinsic and acquired resistance to these novel agents are occurring and the current focus is on developing strategies to overcome these mechanisms of resistance.

Other putatively clinically actionable alterations identified by Robinson et al. included aberrations in the PI3K pathway (49%), DNA repair pathway (19%), MAP kinases (3%), cell cycle pathways (7%), and the WNT pathway (5%) [1]. A cross analysis with primary prostate cancer samples was performed, finding AR and GN17AS mutations to arise exclusively in mCRPC patients.

A later study performed whole generation sequencing in 197 mCRPC samples. They defined eight distinct genomic clusters including a microsatellite instability (MSI) subgroup with high tumor mutational burden associated with mismatch repair deficiency, homologous recombination deficient (HRD) tumors involving genetic alterations in BRCA-related genes, a tandem duplication genotype associated with biallelic loss of CDK12, and a chromothripsis-enriched group [12].

## 3. Current Treatment Based on Next-Generation Androgen Receptor Inhibition in Prostate Cancer

The primary axis of prostate cancer development is the androgen receptor (AR) signaling system, which controls the expression of genes involved in cellular proliferation, differentiation, and prostate cancer cell survival. Androgen deprivation therapy (ADT), which lowers circulating testosterone to castration levels and provides disease control, has long been the go-to treatment for metastatic prostate cancer [13]. Castration resistance develops over time despite ADT. Castration resistance is determined by biochemical progression (three consecutive increases in prostate-specific antigen [PSA] spaced by one week that result in two 50% increases over the nadir, with PSA >2 ng/mL) or radiological progression (the presence of two or more new bone lesions on a bone scan or the enlargement of a soft tissue lesion using serum testosterone levels below 50 ng/dL or 1.7 nmol/L) [14]. The role of these ARSI has expanded beyond non-metastatic or mCRPC to metastatic hormone sensitive prostate cancer (mHSPC) [15,16,17,18,19,20], and, even more, in high risk non-metastatic prostate cancer. Overall, the blockade of the AR axis either by the suppression of androgen synthesis (abiraterone) or the inhibition of AR by second generation AR antagonists (enzalutamide, darolutamide, apalutamide) is key in different settings [21].

### 3.1. Abiraterone

Abiraterone is a selective cytochrome P450 (CYP)17 inhibitor (CYP17A1) that permanently inhibits the manufacture of androgen in the testicles, adrenal tissue, and tumor cells. Due to reduced endogenous glucocorticoid synthesis, prednisone must be administered concurrently in the mCPRC context. In both docetaxel-pretreated (COU-AA-301) and chemotherapy-naive (COU-AA-302) mCRPC patients, two phase 3 trials have shown the benefit of abiraterone (Table 1) [22,23,24,25]. Various trials have investigated the function of abiraterone in mHSPC. De novo high risk mHSPC patients having at least two aggressive characteristics such as a Gleason score more than 8, at least three bone metastatic lesions, and/or visceral metastases were included in the LATITUDE study. PFS and OS both saw a substantial improvement with the addition of abiraterone to ADT (Table 1). Overproduction of mineralocorticoids was the cause of the majority of adverse events (AEs), which included hypertension, nausea, edema, and hypokalemia. However, other toxicities, most of which were grade 1–2, were also reported including fatigue, hot flushes, diarrhea, vomiting, and abnormal liver function [18]. In several clinical contexts, the STAMPEDE Protocol platform assessed the interaction of ADT with abiraterone acetate and enzalutamide. The ADT + abiraterone acetate doublet had no discernible anticancer efficacy in individuals with mHSPC and high risk prostate cancer, although it significantly increased toxicity (Table 1) [21,26]. Finally, the PEACE-1 study, which was focused on the de novo mHSPC situation, randomized 710 patients in a 2 × 2 factorial design to assess the impact of abiraterone in addition to ADT and docetaxel (Table 1) [16]. The trial indicated that the triplet treatment subgroup of individuals with large volume illness improved by 19 months in OS and 30 months in rPFS.

### 3.2. Apalutamide

An oral nonsteroidal antiandrogen called apalutamide (ARN-509) binds specifically to the AR’s ligand-binding domain and inhibits the AR from translocating, binding to DNA, or mediating transcription. Apalutamide has shown benefits in metastasis free survival (MFS) in the SPARTAN trial in nmCPRC. This study included patients with high risk for disease progression (PSA level of ≥8 ng/mL or a PSA doubling time of ≤10 months) (Table 1) [27]. MFS was 40.5 months in the apalutamide group compared to 16.2 months in the placebo group (HR for metastasis or death was 0.28; 95% CI 0.23–0.35; *p* < 0.001). Apalutamide also proved to be beneficial in all secondary end points such as OS with a median increase of 14 months [28]. The most frequent AE were rash (23.8% vs. 5.5%), hypothyroidism (8.1% vs. 2.0%), and fractures (11.7% vs. 6.5%), being the majority grade 1 or 2. In the mHSPC setting, apalutamide was studied in the phase III TITAN trial with a significant improvement in the co-primary endpoints of radiographic PFS and OS (Table 1) [15,29]. The addition of apalutamide has also been evaluated in first line mCRPC in combination with abiraterone and prednisone in a phase III trial (ACIS; NCT02257736) (Table 1) [30]. The primary outcome of rPFS was achieved with no clear impact on OS. Thus, we still need to define whether there is a patient subgroup that could potentially benefit from this combination.

### 3.3. Darolutamide

Darolutamide (ODM-201) is a new generation AR inhibitor with higher AR affinity than enzalutamide or apalutamide, blocking nuclear translocation of the AR receptor. Its main characteristic is that its structure differs from AR antagonists such as enzalutamide or apalutamide, leading to a differential profile of AE as it does not penetrate through the blood–brain barrier [31]. The ARADES phase 1–2 trial was designed to assess the safety and tolerability of this molecule in mCRPC patients [32]. After showing a favorable safety profile, antitumor activity and decrease in PSA levels, clinical development started in men with non-metastatic CRPC (nmCRPC) (ARAMIS trial) and metastatic hormone-sensitive PC (ARASENS trial) (Table 1) [17,33]. ARAMIS was a phase 3 trial that involved 1509 nmCRPC patients with a PSA doubling time of 10 months or less [33]. The primary endpoint, MFS, was achieved at 40.4 months in the darolutamide group and 18.4 months in the placebo group (the darolutamide group’s HR for metastasis or death was 0.41; 95% CI 0.34–0.50; *p* = 0.001). The darolutamide group outperformed all secondary objectives including OS, time to pain progression, time to first symptomatic skeletal event, and time to next cytotoxic treatment. The majority of the grade 1–2 adverse events (AE) in both groups had a comparable frequency (83.2% vs. 76.9%). Fatigue was the most frequent AE, and fracture frequency was similar in both groups. In the ARASENS study, the addition of darolutamide to ADT and docetaxel significantly improved the patient survival in those with de novo mHSPC [17].

### 3.4. Enzalutamide

Enzalutamide is a second-generation, nonsteroidal AR inhibitor that binds to the AR with greater affinity than bicalutamide, impairs AR nuclear translocation, and prevents the recruitment of AR cofactors [34]. Its efficacy was initially evaluated in the post-docetaxel and chemo-naïve setting of mCRPC patients included in the AFFIRM and PREVAIL trials, respectively (Table 1) [35,36]. Safety and efficacy was proven in patients over 75 years and in those with visceral metastasis [37]. The most frequent adverse event (AE) was fatigue, hypertension, diarrhea, hot flashes, and headache; a small proportion of seizures in the enzalutamide group was described (0.6%). In the nmCRPC setting, enzalutamide was tested in the PROSPER phase III trial, showing a significant improvement not only in the primary endpoint of metastases free survival, but also in OS (Table 1) [38]. In the mHSPC setting, the role of enzalutamide has been evaluated in two different clinical trials: the ARCHES and ENZAMET studies, showing a benefit from the addition of enzalutamide to ADT over ADT alone or in combination with standard nonsteroidal antiandrogen therapy, respectively (Table 1) [19,20,39,40].

**Table 1:** Main clinical trials evaluating the role of currently approved androgen receptor signaling inhibitors (ARSI).

Clinical Trial	Setting and Median Follow Up (Months; m)	Number of Patients	Treatment Arms	PFS (Median Months; HR 95%CI)	OS (Median Months) (HR; 95%CI)
ENZALUTAMIDE (ENZA)					
AFFIRM [41]	mCRPC post-doc (14.4 m)	1199 (2:1)	ADT + ENZA vs. PBO	8.3 vs. 2.9 (0.40; 0.35–0.47)	18.4 vs. 13.6 (0.63; 0.53 to 0.75)
PREVAIL [36]	mCRPC (12 & 22 m)	1717 (1:1)	ADT + ENZA vs. PBO	20.0 vs. 5.4 (0.19; 0.15–0.23)	35.3 vs. 31.3 (0.77; 0.67–0.88)
PROSPER [38,42]	NM CRPC (48 m)	1401 (2:1)	ADT + ENZA vs. PBO	MFS = 36.6 vs. 14.7 (0.29; 0.24–0.35)	67 vs. 56.3 (0.73; 0.61–0.89)
ENZAMET [20]	mHSPC (34 m & 40 m)	1125 (1:1)	ADT + TSAA +/− ENZA+/− DOC	3-y-PFS = 68% vs. 41% (0.40; 0.33–0.49)	NR vs. 73.2 (0.70; 0.58–0.84)
ARCHES [19,39]	mHSPC (14.4 m & 44.6 m)	1150 (1:1)	ADT + ENZA vs. PBO (+/− DOC)	49.8 vs. 38.9 (0.63; 0.52–0.76)	NR vs. NR (0.66; 0.53–0.81)
DAROLUTAMIDE (DARO)					
ARAMIS [33]	NM CRPC (29 m)	1509 (2:1)	ADT + DARO vs. PBO	MFS = 40.4 vs. 18.4 (0.41; 0.34–0.5)	NR vs. NR (0.69; 0.53–0.88)
ARASENS [17]	mHSPC (43.7 m)	1305 (1:1)	ADT + DOC +/− DARO	-	NR vs. 48.9 (0.68; 0.57–0.8)
APALUTAMIDE (APA)					
SPARTAN [28]	NM CRPC (52 m)	1207 (2:1)	ADT + APA vs. PBO	MFS = 40.5 vs. 16.2 (0.28; 0.23–0.35)	73.9 vs. 59.9 (0.78; 0.64–0.96)
TITAN [39,40]	mHSPC (22.7 m & 44 m)	1052 (1:1)	ADT + APA vs. PBO (+/− DOC)	NR vs. 22.1 (0.48; 0.39–0.60)	NR v 52.2 (0.65; 0.53–0.79)
ABIRATERONE ACETATE/PREDNISONE (AA/P)					
COU-AA-301 [22,25]	mCRPC post-doc (20.2 m)	1195 (2:1)	ADT + AA/P vs. PBO/P	5.6 vs. 3.6 (0.66; 0.58–0.76)	15.8 vs. 11.2 (0.74; 0.64–0.86)
COU-AA-302 [23,24]	mCRPC (22.2 m & 49.2 m)	1088 (2:1)	ADT + AA/P vs. PBO/P	16.5 vs. 8.2 (0.53; 0.45–0.62)	34.7 vs. 30.3 (0.81; 0.70–0.93)
ACIS [30]	mCRPC (54.8 m)	982 (1:1)	ADT + AA/P +/− APA	24.0 vs. 16.6 (0.70; 0.60–0.83)	36.2 vs. 33.7 (0·95; 0·81–1·11)
LATITUDE [18]	mHSPC (30.4 & 51.8 m)	1209 (1:1)	ADT + AA/P vs. PBO	NR vs. 34.7 (0.62; 0.51–0.76)	53.3 vs. 36.5 (0·66; 0.56–0.78)
PEACE-1 [16]	mHSPC (36 m/45.6 m)	710 (1:1)	ADT + DOC +/− AA/P (+/− RT)	54 vs. 24 (0.50; 0.40–0.62)	NR vs. 52.8 (0.75; 0.59–0.95)
STAMPEDE (Arm G) [43]	mHSPC (40 m)	1917 (1:1)	AA/P vs. PBO	3-y-FFS = 75% vs. 45% (0.29; 0.25 to 0.34)	3-y-OS = 83% vs. 76% (0.63; 0.52–0.76)
STAMPEDE (Arm G & J) [26]	mHSPC (95.8 m/71.7 m)	1003 (1:1)	ADT +/− AA/P +/− ENZA	-	ADT + AA/P + ENZA (0.65; 0.55–0.7) ADT + AA/P (0.62; 0.53–0.73)
STAMPEDE (Arm G & J) [21]	NM high risk PC (72 m)	1974 (1:1)	ADT +/− AA +/− ENZA	MFS = 0.53 (0.44–0.64)	0.60 (0.48–0.73)

PFS: progression free survival; OS: overall survival; mCRPC: metastatic castration resistance prostate cancer; post-doc: post-docetaxel; ADT: androgen deprivation therapy; NM CRPC: non-metastatic castration resistance prostate cancer; MFS: metastasis free survival; mHSPC: metastatic hormone sensitive prostate cancer; 3-y-PFS: 3 years progression free survival; NR: non reached; PBO: placebo.

## 4. Molecular Events on Resistance Mechanisms to AR Signaling Pathway Inhibition

Despite the initial response to ADT in the hormone-naïve setting, which usually achieves rapid decline in PSA levels, this therapy does not completely wean tumor cells of androgens because prostate cancer cells can employ alternative routes to generate AR ligands [44]. Additionally, under the selective pressure from hormonal treatment, prostate cancer cells rely on different steroid hormone receptors including the glucocorticoid receptor (GR) [45] and the progesterone receptor (PR) [46]. During the course of ADT/antiandrogen treatment, AR develops amplifications that lead to AR overactivation as well as point mutations causing ligand promiscuity [47]. Other genomic aberrancies involving the AR, but also coregulator complexes, the selection of AR spicing variants (AR-V), intratumoral steroid hormone synthesis, or posttranscriptional AR regulation are well-known events that occur during ADT/antiandrogen treatment, leading to castration resistance and, eventually, disease progression [48]. Understanding the underlying molecular mechanisms driving tumors to castration resistance has revealed the continuous role of AR signaling in metastatic CRPC. Furthermore, it has set the background for the development of novel therapeutic approaches.

### 4.1. Structural Changes in AR (Figure 1)

#### 4.1.1. Amplification/Overexpression

Amplification of the AR has been found in up to 30% of patients with CPRC, being virtually non-existent in hormone-sensitive disease [49]. Amplification may be due to the selective overgrowth of PC cells [50]. Chen et al. found that the overexpression of AR mRNA in prostate cancer xenograft models was sufficient to explain the transition from a hormone-sensitive to a hormone-refractory stage [51]. Overall, the consequence of AR amplification and/or overexpression is to enhance the PC cells’ sensitivity to low levels of androgens, which leads to disease progression [52].

**Figure 1:**
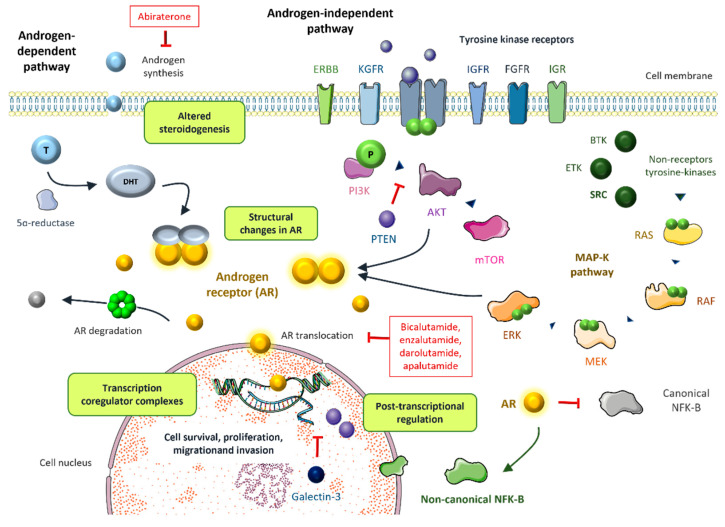
The most relevant mechanisms of resistance to androgen targeted therapy including androgen-dependent and androgen-independent pathways [6,9,11].

#### 4.1.2. Mutations

Several mutations have been identified within the AR gene, predominantly in the ligand-binding domain (LBD), which occur in 15–20% of CRPC. The same as in AR amplification, rather than conveying constitutive activation, gain of function mutations lead to increased AR activity in the presence of low levels of androgens. Two patients with an AR T877A mutation experienced a drastic fall in PSA levels upon withdrawal of the antiandrogen, suggesting thar this mutation may play a role in antiandrogen withdrawal syndrome [53]. Point mutations have also been associated with resistance to novel hormonal agents. For instance, the AR F876L mutation was found to drive resistance to enzalutamide by conferring an antagonist-to-agonist switch [54]. The double mutation F877L/T878A also converts enzalutamide into an AR agonist. Interestingly, this can be overcome by the action of another next-generation antiandrogen such as darolutamide [55]. Moreover, two recent studies have shown that structural disturbances of AR enhancers may decrease sensitivity to hormonal treatments and precipitate castration resistance. In the first study, Takeda et al. characterized a somatically acquired enhancer of AR expression that is activated in the metastatic setting. They demonstrated that a knock-in of a single additional copy of this enhancer was sufficient to foster proliferation in a low-androgen milieu and confer resistance to enzalutamide. Interestingly, disruption of this enhancer caused decreased proliferation by suppressing AR levels [56]. Viswanathan and colleagues analyzed 23 biopsies and cell-free DNA from 86 patients with mCRPC, finding complex rearrangements of the AR locus in most cases, particularly recurrent tandem duplications of an AR upstream enhancer in up to 70–87% of cases, compared to <2% of primary tumors [57]. Targeting epigenetics could be a therapeutic avenue worth exploring in mCRPC.

#### 4.1.3. AR Splice Variants

AR splice variants (AR-Vs) are truncated forms of the AR protein that lack the C-terminal LBD but still have an intact N-terminal domain and a partial or complete DNA binding domain (DBD). These proteins are constitutively active and can activate AR signaling pathways by activating AR targeted genes without the need for ligands [58]. There have been reports of up to 22 AR-Vs including ARV1, ARV7, ARV 567, and ARV8 [59]. Second-generation AR signaling inhibitors such as abiraterone and enzalutamide [60] are resistant to AR-Vs, either as primary resistance in up to 20–40% of cases or as secondary resistance in almost all ones [61]. This is likely due to the fact that both abiraterone and enzalutamide work by binding to the LBD, which AR-Vs lack. The most well-described AR-V is still AR-V7. In CRPC, a recent meta-analysis revealed that AR-V7-positive is linked to greater ECOG performance, metastatic spread, higher Gleason scores, and worse pain management [62].

AR-Vs are transcription factors that join co-regulators to bind DNA and carry out their downstream genomic activities. HOXB13 and AR-V7 interact to activate target oncogenes. It is interesting to note that suppressing HOXB13 in AR-V7-expressing cells prevents AR-V7 from having an oncogenic effect [63]. It is difficult to attempt to inactivate AR signaling since AR-V7 lacks a ligand-binding domain.

This indicates that RNA splicing is essential for the expression of AR-V7 [59]. The RNA helicase DDX39, which is ATP-dependent, controls the expression of AR-V7 and is involved in RNA splicing. The AR-V7 was downregulated in cells that expressed the AR variant after DDX39 was knocked down [64]. An RNA-binding protein called LIN28 is involved in prostate cancer AR signaling. AR mutations and treatment resistance increase as a result of LIN28 overexpression. However, blocking LIN28 can enhance prostate cancer cells sensitive to antiandrogens [65]. Overexpression of a-methylacyl-CoA racemase (AMACR) in prostate cancer has become an immunohistochemical marker.

Dual treatment with docetaxel and an AMARC inhibitor decreased AR-7 expression probably via downregulating HSP27, a cytoprotective chaperone protein that ensures AR stability [66]. In a recent study, castration in mice and prostate cancer cell models led to the activation of NF-KB and the subsequent increase in AR-V7 expression. The NF-KB inhibitor dimethylaminoparthenolide downregulated AR-V7, delaying castration resistance [67]. After depletion of AR-V7, vasopressin 1A receptor (AVPR1A) was found to be the most downregulated gene. Therefore, suppressing APVR1A may decrease cell proliferation [68]. In an AR-V7 enriched environment, there is an overexpression of MAO-A, which results in the alteration of hypoxia-inducible factor-1α signals, contributing to enzalutamide resistance. Targeting MAO-A with antidepressants could restore sensitivity to enzalutamide [69]. As stated by Wang et al. [70], other agents have been identified as AR-V7 inhibitors in CRPC models such as the fatty acid synthase inhibitor IPI-9119, lutelolin, triterpenoid anti-oxidant drugbardoxolone methyl(CDDO-Me), and gonadotropin-releasing hormone (GnRH) antagonist degarelix [71,72,73,74].

Targeting AR-V7 degradation could potentially entail clinical relevance. HSP70 is a shock protein family member that interacts with ubiquitin ligase STUB1. Suppression of HSP70 reverted enzalutamide resistance by decreasing the AR-V7 levels [75]. Xu and colleagues showed that AR-V7 positively regulates E2F1 expression in docetaxel and enzalutamide resistant prostate cancer cells. Auranofin, an FDA-approved drug for the treatment of rheumatoid arthritis, is known to promote AR protein degradation. Auranofin was able to circumvent castration resistance both in vitro and in vivo by downregulating the E2F1-AR3 axis, setting the molecular rationale for drug development in this context [76]. Antimalarial agent bruceantin inhibits both AR full length (AR-FL) and AR-V7 activity by disrupting the interaction of HSP90 with AR-FL/AR-V7, leading to their degradation by the proteasome [77]. Nobiletin, a polymethoxylated flavonoid, induced proteasomal degradation of AR-V7 in vitro, enhancing the sensitivity of AR-V7 positive cells to enzalutamide [78]. Other agents including BCL2 protein inhibitor ABT263 or AKR1C3 inhibitor indomethacin have shown to increase the proteasome-dependent degradation of AR-V7 [79,80].

### 4.2. Coregulator Complexes

The transcriptional function of AR is controlled by over 150 co-regulators [81]). These molecules either enhance (co-activators) or suppress (co-repressors) transcriptional activity. They have a variety of roles including RNA splicing, the recruitment of transcriptional machinery or the modulation of other proteins in the transcriptional complex by phosphorylation, methylation, or ubiquitination [82,83]. The increased activity of co-activators and diminished influence of co-repressors facilitate castration resistance.

Co-activator FKBP51 is a Hsp90 cochaperone that has been proven to be upregulated in castrated mice. This leads to the formation of a superchaperone complex that associates with AR, stimulating androgen binding [84]. Interestingly, patients with a high expression of AR-V7 have increased FKBP51 levels [85]. Depletion of either FKBP51 or FKBP52—another AR co-activator—by using specific inhibitors, reduced the AR dimer formation [86,87].

The steroid receptor coactivators (SRC) enhance AR-induced transcription in a hormone-dependent fashion. The SCR family contains three homologous members, SRC1, SRC2, and SRC3, that have been associated with prostate cancer progression [88] (Figure 1). Under the influence of IL-6, SRC1 phosphorylation leads to AR activation [89,90]. Disrupting the interaction between SRC1/2 and the AR inhibits AR activity in castration-resistant prostate cancer cells [91]. Additionally, miR-137 selectively depleted the expression of SRC1, SRC2, and SRC3 in prostate cancer cells, supporting the potential role of epigenetic targeting in prostate cancer [92].

CBP and p300 are paralogous histone acetyltransferase proteins that precipitate AR activation. Perturbation of p300/CBP function decreases AR activity and reduces tumor cell growth in prostate cancer models [93,94]. CCS1477, a new selective inhibitor of the p300/CBP bromodomain, blocks AR and AR-Vs signaling in the cell line, patient-derive xenografts [95], and in serial tumor biopsies from an ongoing phase I clinical trial [96].

Galectin-3 has been proven to enhance AR transcriptional activity, leading to the overexpression of several AR-target genes such as KLK3 and TMPRSS2. This supports the role of galectin-3 as a potential target molecule in CRPC [97].

### 4.3. Altered Steroidogenesis

Castration resistance develops within a low circulating androgen environment. However, CPRC models show high levels of intra-tumoral androgens. This supports the existence of alternative androgen production involving the adrenal glands but also the prostate cancer cells. Prostate tumor cells overexpress enzymes responsible for androgen synthesis. This allows for the conversion of adrenal precursors such as dehydroepiandrosterone (DHEA) to dihydrotestosterone (DHT) [98]. In the absence of ADT, testosterone acts an intermediary to transform androstenedione (AD) into DHT. However, in an androgen deprived environment, 5α-dione serves as the intermediary between AD and DHT, hence bypassing testosterone [99] (Figure 1). Interestingly, intracrine steroidogenesis not only occurs within the primary prostate cancer, but also in distant metastases [44]. Abiraterone is an orally active inhibitor of the steroidal enzyme CYP17A1, blocking the synthesis of androgens in the adrenal glands as well as in the tumor. A recent study showed that prostate cancer cell lines exposed to abiraterone accumulate steroidogenesis substrates. These precursors are able to activate AR, independent of CYP17A1-mediated conversion into testosterone, leading to cell growth. Interestingly, the potent AR antagonist RD162 was able to reverse proliferation. This supports the rationale for combining CYP17A1 inhibitors (i.e., abiraterone) with potent antiandrogens to suppress AR activation mediated by steroid substrates [100]. The enzyme 3βHSD1 regulates steroidogenesis and abiraterone metabolism and it may be responsible for resistance to abiraterone and enzalutamide [101]. A gain of function mutation of this enzyme has recently been identified as a negative predictive biomarker for response to novel antiandrogens and as a biomarker of poor prognosis [102,103]. Biochanin A is able to overcome resistance to novel antiandrogens by inhibiting 3βHSD1. Daidzein, a biochanin A analogue, showed meaningful PSA reduction in patients with abiraterone-resistant metastatic CRPC [104].

### 4.4. Posttranscriptional Regulation

Posttranscriptional regulation of AR through acetylation, phosphorylation, methylation, SUMOylation, and ubiquitination enhances AR signaling, which leads to cell growth and survival [105]. Arrest-defect-1 protein (ARD1) is an acetyltransferase that forms a complex with AR and Hsp90, leading to AR acetylation, resulting in AR nuclear translocation, AR target gene expression, and prostate cancer tumorigenesis [106]. In vitro and in vivo models have shown that ARD1 activates AR, and ARD1 knockdown inhibits the nuclear translocation of AR [107]. AR phosphorylation at serine 81 (S81) is associated with AR re-activation [108], which is sustained by CDK1 and CDK9, supporting the potential role of CDK inhibitors in the castration resistant setting [109]. Recently, a methylation signature revealed that patients with hypermethylation of AR-associated genes had low AR activity and worse prognosis after ADT [110]. SUMOylation refers to the binding of small ubiquitin like modifiers (SUMO) to AR, typically repressing AR transcriptional activity. An imbalance between SUMOylation and deSUMOylation can lead to tumor progression [111]. Finally, AR ubiquitination induced by different ER ubiquitin ligases can modulate the expression of AR target genes [112]. Siah2 and RNF6 enhance the expression of AR target genes by local turnover of AR and the recruitment of AR co-activators, respectively [113,114], and SPOP represses AR target gene expression by promoting the degradation of AR [115] (Figure 1).

### 4.5. Androgen-Independent Activation

Multiple cytokines, growth factors, and kinase pathways activate AR signaling in a ligand-independent fashion, leading to castration resistance.

The non-receptor tyrosine kinase Src fosters the proliferation of prostate cancer cells through activation of the MAPK cascade. In fact, Src kinase activation is associated with androgen-independent cell growth and tumor invasion [116,117]. Although Src inhibitors have been successful in preclinical models [118], their performance in clinical trials has been disappointing thus far [119]. Other non-receptor tyrosine kinases such as Btk and Etk have been targeted in recent years [120,121] (Figure 1).

The PI3K/AKT/mTOR pathway also plays an important role. Almost all metastatic prostate cancer show loss of PTEN, which is an inhibitor of this cascade. The activation of the downstream effectors of the PI3K/AKT/mTOR pathway leads to castration resistance [122] (Figure 1). Interestingly, this pathway interacts with AR signaling through several reciprocal inhibitory loops [123]. The AKT inhibitor capivasertib, in addition to docetaxel, improved outcomes in a phase II trial with CRPC patients, particularly in patients that had received drugs targeting the androgen receptor [124]. A phase III trial of capivasertib and abiraterone vs. placebo and abiraterone in PTEN deficient mHSPC is ongoing (CAPItello-281, NCT04493853) [125]. As discussed above, the AKT inhibitor ipatasertib in combination with abiraterone seems to improve the survival outcomes in PTEN-loss tumors.

NF-kB is a transcription factor that has been associated with prostate cell survival and tumor proliferation [126]. There is a relevant crosstalk between NF-KB and androgen receptor signaling [127]. The AR blocks canonical NF-kB expression but induces non-canonical NF-kB activation [128] (Figure 1). This could be of major importance in driving androgen independence, since the loss of androgen repression of NF-kB target genes is linked to poor prognosis [129]. Inhibition of NF-kB signaling resensitizes castrate-resistant prostate cancer cells to androgen receptor targeted therapies [130]. Additionally, a key role has recently been identified on cancer stemness in prostate cancer cells related to SOX2/OCT4 expression, and the PI3K/Akt downstream signaling has been associated with playing a role in keeping this pathway. This molecular activation has been related to tumor progression irrespective to AR signaling as well as a potential resistance mechanism to therapy [131,132].

Growth factor pathways such as IGF, KGF, or FGF activate AR in a castrate resistance setting. Several RTKs such as EGFR, IGR, or HER-2 can also enhance AR activity (Figure 1). It is known that MYB overexpression plays an important role in androgen-depletion resistance and prostate cancer aggressiveness [133]. Recently, it has been discovered that there is a close interaction between MYB and the AR. MYB-overexpressing prostate cancer cells retain AR in the nucleus, even in androgen-deprived conditions, leading to enhanced transcriptional activity [134].

## 5. Novel Agents under Research That to Overcome Those Resistance Mechanisms

### 5.1. Galeterone

Galeterone is a multitargeted, selective drug that alters androgen signaling in a variety of ways. It works as a powerful AR antagonist and a selective CYP17 inhibitor, inducing an increase in AR protein breakdown and lowering AR expression in prostate cancer cells. Galeterone therapy in prostate cancer models has led to a considerable decrease in the levels of both full-length AR and AR-V7, according to preclinical in vitro and in vivo findings. Galeterone is also effective against AR point mutations T878A, and early findings indicate that it may also successfully target cells with F876L mutations [135]. Galeterone-based Androgen Receptor Modulation Optimized for Response (ARMOR) Phase I and Phase II trials on patients with metastatic and non-metastatic CRPC showed sufficient tolerance and potential efficacy [60].

In the ARMOR1 trial, 49.0% of patients had a 30% drop in PSA and 22.4% had a 50% reduction. These findings led to the development of a phase 3 clinical study (ARMOR3-SV) [136] that focused on the splice variant AR-V7. Patients with mCRPC who exhibited AR-V7 were given the option of receiving enzalutamide 160 mg or galeterone 2550 mg on a random basis. Sadly, the research was stopped after it was determined that its survival endpoints were unlikely to be met.

### 5.2. ODM-204

ODM-204 is a non-steroidal compound constructed as a potent dual inhibitor of both the CYP17A1 enzyme and AR-mediated signaling at the receptor level [6,9,11]. Following favorable outcomes in xenograft models, phase I–II studies were planned in patients with progressive mCRPC [137]. In the DUALIDES study, escalating doses of ODM-204 together with prednisone were administered orally twice a day. ODM-204 was well-tolerated and preliminary antitumor activity was observed in some patients [137]. Unfortunately, pharmacokinetic difficulties have stalled the development of this molecule.

### 5.3. ODM-208

Recently, the phase I/II CYPIDES trial explored ODM-208, a CYP11A1 inhibitor able to suppress the synthesis of all steroid hormones and precursors in heavily pretreated patients with an activating AR LBD mutation. ODM-208 showed meaningful PSA reductions and up to four partial responses in 17 evaluable patients [138].

### 5.4. Proxalutamide (GT-0918)

In contrast to bicalutamide and enzalutamide, proxalutamide is a new non-steroidal AR antagonist that suppresses AR-mediated gene transcription more potently while maintaining quiet antagonism in CRPC cells [139]. Studies have shown that it can downregulate AR protein levels and impede the transcriptional activity of both wild-type and clinically significant mutant ARs. Additionally, it has been discovered to suppress the AR, which suggests that it may be more effective than other second-generation AR antagonists. Currently, this drug is being developed in a phase I multicenter, open-label clinical study for men with progressive mCRPC treated with chemotherapy (docetaxel) and hormone treatment (abiraterone or enzalutamide) (NCT02826772).

### 5.5. Bipolar Androgen Therapy (BAT)

BAT is an emerging approach to androgen inhibition in the mCRPC setting. As already mentioned, CRPC remains dependent on AR signaling. Preclinical models suggest that supratherapeutic testosterone levels could reduce CRPC growth and promote cell death [140]. BAT aims to disrupt the CRPC cells’ ability to adapt to a low-androgen environment by alternating cycles of high-dose testosterone with cycles of androgen inhibition, resulting in the minimum testosterone levels and cell death. RESTORE is a multicohort phase 2 study that includes patients who have progressed to hormone therapy (including enzalutamide or abiraterone). BAT showed clinical activity by decreasing PSA levels, but did not reach statistical significance [141]. A cohort of this study analyzed results from 29 patients after first hormonal treatment excluding patients receiving second generation antiandrogens. In this setting, BAT resulted in PSA and radiologic responses, both in mCRPC and in non-metastatic CRPC. Response to abiraterone and enzalutamide at progression was favorable [142]. The TRANSFORMER trial [143] recruited men with asymptomatic mCRPC progressing on abiraterone and compared BAT treatment with enzalutamide. The results showed no superiority of BAT compared to enzalutamide in terms of PFS and PSA response. However, BAT improved the magnitude and duration of response to enzalutamide after treatment with abiraterone, suggesting again the presence of a sensitizing effect and a potential role for sequential BAT and enzalutamide as a single therapy.

### 5.6. Proteolysis-Targeting Chimeras (PROTACs)

This is a novel technology targeting the degradation of the AR. PROTACs are molecules that work as a trimeric complex between a target protein and an E3 ubiquitin ligase, enabling target ubiquitination and subsequent degradation [144]. ARCC-4 is one of these AR degraders. It also prevents cell proliferation, even in enzalutamide resistant cells, becoming a potential therapeutic option in these patients. PROTAC-mediated AR degradation can overcome AR resistance mechanisms such as AR amplifications, AR mutations, and intra-tumoral androgen synthesis [145]. The phase I clinical trial of ARV-110 [146], an orally bioavailable PROTAC, included 18 patients that had progressed to at least two previous lines of treatment. Anti-proliferative activity was observed including PSA reduction of ≥50% in two patients. Toxicity was manageable with two patients developing grade 3–4 elevated AST/ALT levels. The expansion cohort of the phase I/II clinical trial has shown promising activity in heavily pretreated mCRPC, particularly in patients with AR T878 and/or H875 mutations [147].

### 5.7. Immune Checkpoint Inhibitors

The identification of different mCRPC phenotypes may prove to be clinically relevant. Aside from the agnostic FDA approval of pembrolizumab [148] and dostarlimab for MSI-H or dMMR/TMB-H [149] and for dMMR tumors [150], respectively, there are clinical trials ongoing testing immune checkpoint inhibitors (ICIs) in selected patients with mCRPC. In the KEYNOTE-199 trial, pembrolizumab in PD-L1 positive mCRPC previously treated with docetaxel and endocrine therapy showed a disease control rate (DCR) of 10% and a median OS of 9.5 months [151]. Recently, we learned from the phase III clinical trial KEYNOTE-921, that pembrolizumab does not provide a survival benefit when combined with docetaxel versus chemotherapy alone in unselected mCRPC [152]. Hence, it seems that adequate patient selection is key to finding the subset of patients that could potentially benefit from ICIs. In prostate cancer, MSI-H seems to be a more reliable biomarker than TMB. A prospective comparison between ICI vs. taxane-based therapy showed that patients with a TMB ≥10 mutations per Mb achieved better survival outcomes when treated with immunotherapy [153]. Combination schemes with antiandrogens have also been explored. Unfortunately, the phase III trial IMbassador 250 of enzalutamide plus atezolizumab versus enzalutamide alone was stopped early due to futility [154]. The results of the ongoing KEYNOTE 641 trial of enzalutamide plus pembrolizumab [155] are awaited after promising data from the phase II trial [156]. Interestingly, the combination of apalutamide and anti-PD-1 cetrelimab is being evaluated in small cell neuroendocrine prostate cancer (NCTNCT04926181). Thus, biomarker selection and treatment combination will be key in this setting [157].

### 5.8. PARP Inhibition

PARP inhibitors have recently entered the therapeutic landscape of mCRPC. Olaparib has been approved by regulatory agencies for the treatment of germline or somatic homologous recombination repair (HRR) gene-mutated mCRPC following the results of the phase III PROfound trial [158]. However, a subgroup analysis of this trial showed that for almost all of the HRR mutations other than BRCA, olaparib did not offer a clear benefit. For this reason, the optimal treatment approach in these patients is yet to be elucidated. Additionally, the PARP inhibitor rucaparib has been approved for BRCA-mutated mCRPC based on the results of the TRITON2 trial [159]. Trials exploring the combination of PARP inhibitors with ARSI are currently ongoing. Recently, the PROpel and MAGNITUDE phase III trials exploring the combination of abiraterone with olaparib and niraparib, respectively, proved meaningful rPFS improvement for the experimental arm, along with a trend toward OS in the PROpel study [160,161]. On a similar note, the phase III trial TALAPRO-2 of talazoparib plus enzalutamide versus enzalutamide has achieved its primary endpoint of rPFS [162]. The potential synergy from this combination was assessed in the BRCAAway trial, a phase 2 randomized study in patients with inactivating BRCA1, BRCA2, and/or ATM alterations. The first results presented at the ASCO Congress 2022 showed a significant improvement on the median PFS from the abiraterone acetate and olaparib combination over either agent alone. The possibility to crossover at disease progression will also offer data on sequencing compared with the combination [163].

### 5.9. PI3K/AKT/mTOR and MAPK Pathway Inhibition

As for the PI3K/AKT/mTOR pathway, preclinical studies in mice have established that loss of PTEN promotes resistance to castration [164], hence upregulating the PI3K pathway. Interestingly, inhibition of the PI3K pathway in PTEN negative prostate cancers activates the AR pathway [165]. Since AR and PI3K seem to compensate each other’s inhibition, simultaneous targeting of AR and PI3K has been the proof-of-concept for developing clinical trials in this setting. The phase III clinical trial IPATential150 randomized unselected mCRPC patients to receive the PI3K inhibitor ipatasertib plus abiraterone and prednisolone or the placebo plus abiraterone and prednisolone. Although the primary endpoint of PFS was not met in the intention-to-treat population, ipatasertib improved PFS in PTEN-loss tumors [166]. The phase III trial Capitello280 in mHSPC is ongoing [167]. As for cell cycle targeting in mCRPC, a phase Ib/II clinical trial with ribociclib and docetaxel in ARSI-pretreated mCRPC, showed a favorable toxicity profile and encouraging results (median rPFS of 8.1 months) [168]. Clinical trials with drugs targeting MAPK downstream proteins (i.e., BRAF/MEK inhibitors) or the WNT pathway (i.e., porcupine inhibitors) are yet to be designed.

### 5.10. Cell Cycle Pathway Inhibition

Approximately 20% alterations in cell cycle genes have been identified in CRPC with RB1 loss, CCND1 amplification, and other alterations in CDKN2A/B, CDKN1B, and CDK4 as the main genes affected [1]. Preclinical data have demonstrated the ability from abemaciclib to enhance effective senescence in prostate cancer cells expressing AR. These data have also been obtained with AR+ Prostate 22RV1 Xenograft models showing a tumor reduction with abemaciclib treatment [169]. Therefore, clinical trials have been designed and are currently ongoing with CDK4/6 inhibitors in monotherapy [170,171], but also in combination [172,173,174,175,176] in prostate cancer.

### 5.11. Radionuclides

Different attempts have been reported trying to combine Radium223 with new hormone agents. The most relevant data came from the prospective phase III ERA trial in which the addition of abiraterone acetate plus prednisone/prednisolone to Radium223 reported an earlier and higher rate of SRE (HR 1.12 [95% CI 0.917–1.374]; *p* = 0.2636) than with Radium223 monotherapy [177]. Based on this increase in bone fractures and death, some regulatory agencies such as the European Medicines Agency in July 2018 decided to modify the recommendation in the use of Radium223 to patients that had received two previous regimens in the metastatic setting or to patients that were not candidates for other treatments. This restriction raised relevant issues such as the adequate identification of those patients unfit for chemotherapy and/or novel hormone agents, the concern regarding bone health maintenance, the impact of a prolonged use of corticoids, and how to optimize the therapeutic sequences in patients with mCRPC. The key role of bone protective agents has been demonstrated in the PEACE III trial. Gillessen S et al. reported a reduction in the cumulative risk of fracture at 1.5 years to practically zero with the use of those agents at least 6 weeks before the initiation of Radium223, both in the enzalutamide and the combination group compared with the previously reported risk without antiresorptive agents of 22.3% and 45.9%, respectively [178]. The combination of ARSI with radionuclides is improving as in the mHSPC setting, 177LuPSMA-617 is being combined with ARSI in a phase III clinical trial (NCT04689828).

### 5.12. Continuing AR Blockade upon Progression

As in breast cancer, maintaining hormonal treatment after disease progression is being explored in prostate cancer. The randomized phase II ABIDO trial compared abiraterone plus docetaxel versus docetaxel alone following progression to first line abiraterone. This study was unable to demonstrate a significant clinical benefit of abiraterone maintenance [179]. Surprisingly, the PRESIDE phase III trial, testing enzalutamide in the same clinical setting, did meet its primary endpoint of PFS [180]. It is important to note that the ABIDO trial, but not the PRESIDE study, allowed for the inclusion of patients with primary resistance to ARSI. Additionally, there were some differences between the two trials regarding the consideration of progressive disease to first line ARSI. These factors could, to some extent, explain the different outcomes [181]. Nonetheless, considering the PEACE-1 trial results, the population depicted in the PRESIDE study will not be representative of clinical practice in the near future (Table 2).

## 6. Discussion

Metastatic prostate cancer treatment is initially based on androgen inhibition to reach undetectable testosterone levels. However, recent findings highlight the continuous role of androgen receptor signaling in disease progression in up to 70% of patients whose tumor show different AR aberrations [1]. Indeed, even though patients reach the castration resistance stage, androgen inhibition is still effective with novel antiandrogens that have come in the field such as abiraterone acetate, apalutamide, enzalutamide, or darolutamide [11]. Moreover, other pathways are also involved in this tumor progression such as PI3K/AKT/mTOR pathway activation in 30–40% of patients, DDR gene alterations in 20–30% of patients, cell cycle alterations in 20% of patients, or the Wnt signaling pathway in 18% of patients [1]. Thus, key questions arise as to whether to sequence with a different therapeutic strategy, but with which treatment or combination strategy. This is an important question as prostate cancer has entered the precision medicine era, so current research is moving forward to overcome the primary or acquired resistance mechanisms involved in prostate cancer to allow physicians to improve the strength of their clinical decisions. Multiple drugs are showing activity in metastatic prostate cancer, but the main question remains on how to sequence them in each patient.

As AR signaling plays a key role along the disease of prostate cancer and ARSI has dramatically modified the natural history of this disease in different clinical settings, AR aberrations are still an essential target to inhibit during the continuum of care in prostate cancer.

## 7. Conclusions

Novel drugs with potential activity in patients harboring tumors whose AR-related alterations are resistant to current ARSI, but can be targetable with these new agents, offer an exceptional opportunity to continue with androgen inhibition and, hopefully, survival contribution to patients with metastatic prostate cancer. Alone or in combination with effective therapies will require further answers as research in this field is rapidly increasing.

## Figures and Tables

**Table 2:** Ongoing trials for emerging drugs in prostate cancer.

Drug	Mechanism of Action	Ongoing Clinical Trials	
		HSPC	mCRPC
ANDROGEN SIGNALING INHIBITION			
GALETERONE	*Selective CYP17 inhibitor and a potent AR antagonist*	-	-
ODM-204	*Dual inhibitor of both the CYP17A1 enzyme and AR-mediated signaling at the receptor level*	-	-
ODM-208	*CYP11A1 inhibitor able to suppress the synthesis of all steroid hormones and precursors*	-	NCT03436485
PROXALUTAMIDE (GT-0918)	*Non-steroidal AR antagonist, that inhibits AR- mediated gene transcription*	-	NCT03899467
BIPOLAR ANDROGEN THERAPY (BAT)	*Alternating cycles of high-dose testosterone with cycles of androgen inhibition*	-	NCT04558866, NCT04704505, NCT03522064
PROTACs	*Trimeric complex between a target protein and an E3 ubiquitin ligase, enabling target ubiquitination and subsequent degradation*	-	NCT03888612, NCT04428788
IMMUNE CHECKPOINT INHIBITORS			
PEMBROLIZUMAB	*Immune checkpoint inhibitor*	NCT05568550, NCT04931979	NCT03834519, NCT03506997, NCT05563558, NCT04104893, NCT04090528, NCT02861573, NCT04471974, NCT04221542
*tor*			
*Targeting PD-1*			
NIVOLUMAB	*Immune checkpoint inhibitor*	NCT03637543, NCT04019964, NCT04989946, NCT03543189, NCT04477512, NCT04126070	NCT02933255, NCT04109729, NCT04100018, NCT05502315, NCT05445609, NCT04717154, NCT05169684, NCT05150236
	*Targeting PD-1*		
	*Targeting PD-1*		
ATEZOLIZUMAB	*Immune checkpoint inhibitor* *targeting PD-L1*	NCT04262154	NCT04404140, NCT04751929, NCT05168618, NCT04446117, NCT03673787
PARP INHBITION			
OLAPARIB	*PARP inhibitor*	NCT0474804, NCT03047135, NCT05167175	NCT05501548, NCT04038502, NCT05457257, NCT03317392, NCT03834519, NCT03012321, NCT04951492, NCT05262608, NCT05005728, NCT02861573
NIRAPARIB	PARP inhibitor	NCT04497844, NCT04037254,	NCT04592237, NCT04288687
RUCAPARIB	*PARP inhibitor*	NCT03413995, NCT03533946	NCT04253262, NCT04455750, NCT03442556
TALAZOPARIB	*PARP inhibitor*	NCT04332744, NCT04821622, NCT04734730	NCT04824937, NCT05425862, NCT04846478, NCT04019327, NCT04703920
PI3K/AKT PATHWAY INHIBITION			
IPATASERTIB	*AKT inhibitor*	-	NCT03072238, NCT04404140
CAPIVASERTIB	*AKT inhibitor*	NCT04493853	
CDK INHIBITION (CELL CYCLE)			
PALBOCICLIB	*CDK4/6 inhibitor*	-	NCT04606446
ABEMACICLIB	*CDK4/6 inhibitor*	NCT05288166	NCT05113537, NCT04408924, NCT03706365
